# Bcl-3 promotes TNF-induced hepatocyte apoptosis by regulating the deubiquitination of RIP1

**DOI:** 10.1038/s41418-021-00908-7

**Published:** 2021-12-01

**Authors:** Yiming Hu, Haohao Zhang, Ningxia Xie, Dandan Liu, Yuhang Jiang, Zhi Liu, Deji Ye, Sanhong Liu, Xi Chen, Cuifeng Li, Qi Wang, Xingxu Huang, Yongzhong Liu, Yufang Shi, Xiaoren Zhang

**Affiliations:** 1 Affiliated Cancer Hospital and Institute of Guangzhou Medical University; Key Laboratory for Cell Homeostasis and Cancer Research of Guangdong Higher Education Institutes; State Key Laboratory of Respiratory Disease, 510000 Guangzhou, China; 2 CAS Key Laboratory of Tissue Microenvironment and Tumor, Shanghai Institute of Nutrition and Health, Shanghai Institutes for Biological Sciences, University of Chinese Academy of Sciences, Chinese Academy of Sciences, 200031 Shanghai, China; 3 Institute of Interdisciplinary Integrative Medicine Research, Shanghai University of Traditional Chinese Medicine, 201203 Shanghai, China; 4 State Key Laboratory of Oncogenes and Related Genes, Shanghai Cancer Institute, Renji Hospital, Shanghai Jiao Tong University School of Medicine, 200032 Shanghai, China

**Keywords:** Protein folding, Genetics research

## Abstract

Tumor necrosis factor-α (TNF) is described as a main regulator of cell survival and apoptosis in multiple types of cells, including hepatocytes. Dysregulation in TNF-induced apoptosis is associated with many autoimmune diseases and various liver diseases. Here, we demonstrated a crucial role of Bcl-3, an IκB family member, in regulating TNF-induced hepatic cell death. Specifically, we found that the presence of Bcl-3 promoted TNF-induced cell death in the liver, while Bcl-3 deficiency protected mice against TNF/D-GalN induced hepatoxicity and lethality. Consistently, Bcl-3-depleted hepatic cells exhibited decreased sensitivity to TNF-induced apoptosis when stimulated with TNF/CHX. Mechanistically, the in vitro results showed that Bcl-3 interacted with the deubiquitinase CYLD to synergistically switch the ubiquitination status of RIP1 and facilitate the formation of death-inducing Complex II. This complex further resulted in activation of the caspase cascade to induce apoptosis. By revealing this novel role of Bcl-3 in regulating TNF-induced hepatic cell death, this study provides a potential therapeutic target for liver diseases caused by TNF-related apoptosis.

## Introduction

Enhanced apoptosis of hepatocytes is a typical feature of various liver diseases, such as drug or toxicant-induced injury, viral hepatitis, cholestasis, ischaemia/reperfusion, and liver preservation for transplantation [1–4]. Elucidation of mechanisms that mediate hepatocyte apoptosis is thus urgently needed to develop potent interventions to treat liver diseases. Accumulating evidence has shown that hepatic apoptosis is modulated by a complicated network of apoptotic and inflammatory signals, which are mainly initiated by tumor necrosis factor receptor (TNFR) family members [5, 6], but the underlying mechanism is still largely unknown.

TNF is an intensively studied pro-inflammatory cytokine that plays important roles in cellular survival, proliferation, differentiation and death [7, 8] and is initiated by the engagement of TNF with its receptor TNFR1. The distinct outcomes of TNF signalling rely on the sequential formation of different complexes, named Complex I and Complex II [9, 10]. Upon Complex I formation, TNFR1 facilitates the recruitment of TNFR1-associated death domain, TNFR-associated factor 2/5 (TRAF2/5), receptor interacting protein 1 (RIP1) and cellular inhibitor of apoptosis protein 1/2 (cIAP1/2) [11, 12]. This recruitment is crucial for activating NF-κB, JNK and p38 cascades [13–15] and further promoting the transcription of pro-survival genes. Nevertheless, under certain circumstances when the constitution of Complex I is perturbed, RIP1 dissociates from Complex I and recruits Fas-associated death domain (FADD) and Caspase 8 to form Complex II [10, 11, 16, 17]. Upon engagement of Complex II, activated Caspase 8 initiates caspase signalling to drive cell death [8]. It has been reported that transition from Complex I to Complex II is mainly regulated by the ubiquitination status of RIP1, and deubiquitination of RIP1 facilitates its involvement in the formation of Complex II [11, 16, 18]. Recently, many factors that regulate RIP1 ubiquitination have been revealed, with cIAPs, A20 and CYLD as the primary factors [19–21].

B-cell lymphoma 3 (Bcl-3) is usually recognized as a pro-oncogene, which is highly expressed in the liver [22], but its specific roles in hepatocyte proliferation or apoptosis are still undefined. First identified in patients with B cell chronic lymphocytic leukaemia, Bcl-3 was shown to function as a coactivator or inhibitor with dimers of the NF-κB subunits p50 and p52 to regulate the transcription of NF-κB target genes. Noticeably, Bcl-3 in cancer cells mainly resides in the nucleus, while in hepatocytes, Bcl-3 is mostly expressed in the cytoplasm [23], suggesting that the function of Bcl-3 is cell-type specific. More recently, it was shown that overexpression of hepatic Bcl-3 enhances DEN/PB-induced hepatocyte apoptosis, thus alleviating inflammation during HCC initiation [24]. This finding provides direct evidence that Bcl-3 plays important roles in regulating hepatocyte apoptosis, while currently, there is less information available concerning its role in TNF-induced hepatocyte death.

In the present study, a well-established acute liver injury mouse model with TNF and D-galactosamine (D-GalN, a hepatocyte-specific sensitizer) [25–28] co-stimulation was used to investigate the role of Bcl-3 in hepatocyte apoptosis. The results showed that Bcl-3 deficiency protected both mice and in vitro hepatic cells from TNF-induced apoptosis. This protection was achieved through ubiquitinating RIP1 by interacting with CYLD, which further promoted the formation of Complex II. Indeed, less formation of Complex II and more ubiquitination of RIP1 were detected in Bcl-3-depleted cells with TNF stimulation. Taken together, these findings suggest that Bcl-3 could serve as a potential therapeutic target for liver diseases caused by TNF-induced apoptosis.

## Materials and methods

### Mice

Bcl-3 knockout (KO) mice on C57BL/6 background were generated by Shanghai Bioray Biotech Co., Ltd, using CRISPR/Cas9 technique. Bcl-3 KO mice and their representative wild-type control mice were maintained in specific-pathogen-free facility. All animal experiments were carried out in accordance with the guide for the care and use of laboratory animals and were approved by the Institutional Animal Care and Use Committee of Shanghai Institutes for Biological Sciences, Chinese Academy of Sciences.

Primers used for genotyping: F: 5′-3′ CACACGCACAAATGTGGTACAC; R: 5′-3′ ACCACCACTGCCCATCTTATAG.

### Reagents and antibodies

TNFα (210-TA) (for in vivo experiments) was from R&D system; D-GalN (101743) was from Millipore; ConA, CHX, puromycin and doxycycline (DOX) were from Sigma Aldrich; ZVAD-FMK (s7023) and sp600125 (s1406) were from Selleck; LCL161 (HY-15518) was from MCE; TNFα (300-01 A) (for in vitro experiments) was from Peprotech; Flag-TNF was from Enzo Life (ALX-522-009-C050); Lipofectamine 2000 Transfection Reagent was from Invitrogen. The following antibodies were used for flow cytometry: FITC-Annexin V (556420), 7-AAD (559925) were from BD Biosciences; Alexa Flour^®^ 488 anti-rabbit IgG was from Invitrogen. The following antibodies were used for immunoprecipitation and immunoblotting: anti-Bcl-3 (sc-185), anti-Caspase 8 (sc-6136), anti-STAT3 (sc-482), anti-UB (sc-8017), anti-rabbit IgG (sc-2027) and anti-mouse IgG (sc-2025) were from Santa Cruz; anti-Caspase 8 (9746), anti-cleaved Caspase 3 (9661), anti-pJNK (4668), anti-JNK (9252), anti-pIκB (2859), anti-PARP (9542), anti-FADD (2782), anti-A20 (5630), and anti-CYLD (8462) were from Cell Signaling Technology; anti-HA tag (51064) and anti-MYC tag (60003) were from Proteintech; anti-cIAP1/2 (MAB3400) was from R&D; anti-pSTAT3 (CY6566) was from Abways; anti-RIP1 (610458) was from BD Biosciences; anti-β-Actin (A2228), anti-Flag (F3165) and anti-Flag M2 affinity gel (A2220) were from Sigma Aldrich; anti-GAPDH (KC-5G4).

### Cell lines

Primary MEFs were prepared from embryos at embryonic day 13.5 from Bcl-3^+/−^ mice and immortalized by the 3T3 protocol as described previously [29]. The immortalized human liver cell line LO2 was a kind gift from Dr. Yongzhong Liu (Shanghai Jiaotong University). HepG2, NIH3T3, and HEK293T cells were purchased from the American Type Culture Collection (ATCC). Bcl-3 and CYLD KO cells were generated using CRISPR/Cas9 system. Briefly, oligo encoding gRNAs (Bcl-3-sgRNA1: 5′-CCCGTGCAGATGAGGACGGAGAC-3′, Bcl-3-sgRNA2:5′-CCTCTCCATATTGCTGTGGTGCA-3′; CYLD-sgRNA1:5′-AAGCTCCTTAAAGTACCGAAGGG-3′, CYLD-sgRNA2:5′-AAGTACCGAAGGGAAGTATAGG-3’) were constructed into the PGL3-U6-EGFP vector. The target cells were co-transfected with LentiCas9-Blast and PGL3-EGFP-sgRNA plasmids for 48 h and subsequently screened by FACS for GFP positive mono-clone cells into 96-well plate. After 3–4 weeks, the cells checked for KO efficiency were used for the subsequent experiments. All cells were all grown in Dulbecco’s Modified Eagle’s Medium (DMEM, Hyclone, Logan, UT, USA) supplemented with 10% FBS, 100 μg/ml streptomycin and 100 U/ml penicillin. Cells were maintained at 37 °C in a 5% CO_2_ incubator.

### Plasmids and lentivirus-delivered gene overexpression and knockdown

Flag-tagged Bcl-3 was cloned into the pLVX-IRES-Puro plasmid (Clontech 632183), a lentiviral expression vector, to construct Bcl-3 overexpression plasmid. Two different Bcl-3 shRNA expression sequences (V3THS-407972, V3THS-356488) were cloned into a tet-on expression vector pTRIPZ (Open Biosystems) to construct Bcl-3 knockdown plasmids. The non-silencing lentiviral shRNA vector was conducted as the control. CYLD shRNA expression sequence was cloned to pLVX-IRES-Puro plasmid, the target sequence was: 5′-gatccGCCTCATGCAGTTCTCTTTGTTCAAGAGACAAAGAGAACTGCATGAGGTTTTTTACGCGTg-3′. The constructs above were co-transfected with plasmids pMD2G and psPAX2 (Addgene) into Lenti-x cells to harvest lentivirus supernatants after 48 h. To obtain stable Bcl-3 knockdown cell lines, lentivirus infected LO2 and HepG2 were cultured with 3 μg/ml puromycin in the medium for 1 week, following by treated with 1 μg/ml doxycycline (DOX) for more than 3 days. To overexpress Bcl-3 in NIH3T3, lentivirus supernatant was added to the culture medium for 48 h. Flag-tagged-Bcl-3 was cloned into pcDNA3.1. Myc-tagged-CYLD was cloned into pcDNA3.1. HA-tagged-ubiquitin (Addgene plasmid 17608) was a kind gift from Dr. Sandra Weller. HA-Flag-tagged-RIP1 was a kind gift from Dr. Haibing Zhang (SIBS, CAS).

### Induction of acute hepatitis

To prepare mice for TNF/D-GalN induced acute hepatitis, 10- to 12-week-old male Bcl-3 KO and age-matched male WT mice, were given an intraperitoneal injection of D-GalN (700 mg/kg), followed by intraperitoneal injection of TNF (10 μg/kg) thirty minutes later. ConA (20 mg/kg) was intravenously injected to induce hepatitis. Survival was monitored every 2 h until 2 or 3 days. Mice in some experiments were sacrificed at indicated times for liver sample collection and processed for serum ALT (JianCheng Bioengineering Institute) detection or immunohistochemical analysis.

### Bone marrow transfer assay

Four to six-week-old recipient Bcl-3 KO and WT mice were lethally irradiated by 850 cGy, and then the recipient mice were injected via the tail vein with 4 × 10^6^ bone marrow cells from donor Bcl-3 KO or WT mice. Four groups of chimeras were conducted: WT → WT, WT → KO, KO → WT and KO → KO. After transplantation, the mice were given with drinking water containing neomycin (2 g/L) for 2 weeks. Eight weeks after bone marrow reconstitution, the transplanted mice were applied to acute hepatitis as described above.

### Apoptosis analysis by flow cytometry

Apoptosis was detected by Annexin V/7-AAD staining. In brief, after wash with ice cold PBS, harvested cells were resuspended in 100 μl binding buffer (10 mM Hepes (pH 7.4), 140 mM NaCl and 2.5 mM CaCl_2_) containing 5 μl Annexin V and 5 μl 7-AAD and incubated in the dark for 20 min at room temperature. Binding buffer was added to each sample for subsequently flow cytometric analysis. Flow cytometric analysis was carried out with Gallios Flow Cytometer (Beckman Coulter) and FlowJo software (Tree Star Inc).

### Immunochemistry

For histological analysis, target tissues were fixed in 4% paraformaldehyde, embedded in paraffin, and then sectioned into 5 μm slides for subsequently staining with hematoxylin and eosin (H&E). To detect the cleaved Caspase 3, sections were performed using standard protocols. Briefly, the slides were deparaffinized and immersed in 3% H_2_O_2_ for thirty minutes, followed with antigen retrieval and 5% goat serum (Jackson, NIS-0083) blocking, and then incubated with diluted anti-cleaved Caspase 3 (Cell Signaling, 9661) antibody. TUNEL assay was performed using a TUNEL cell death detection kit (Beyotime Co.) according to manufacturer’s instructions. Images were photographed by a general optical microscope (Carl Zeiss) and analyzed using Image-Pro Plus 6.0 software (Media Cybernetics Inc).

### RNA extraction and RT-PCR

Total RNA of tissues or cells were extracted using TRIzol Reagent (Invitrogen, 15596-018) following manufacturer’s instructions. The RNA was reverse transcribed into cDNA using PrimeScript^TM^ RT Master Mix (Takara, RR036A). After transcription, RT-PCR was performed by ViiA 7 Real-Time PCR System (Life Technologies) using SYBR Premix Ex Taq (Takara, RR420A) in accordance with the manufacturer’s instructions. Each sample was performed in triplicate. The primers used for detection were described in Supplementary Table 1.

### Western blot analysis

Liver tissues or cells were washed with PBS before ruptured in ice-cold NP-40 buffer (Beyotime, P0013F) supplemented with protease inhibitors cocktail (Roche Diagnostics, 4693116001), phosphatase inhibitor cocktail (Roche Diagnostics, 4906845001) and PMSF (Beyotime, ST506). The lysates were incubated on ice for 30 min before clarified by centrifugation at 13,000 *g* for 15 min, and then the supernatants were collected after centrifugation. The concentrations of total protein samples were estimated using BCA protein assay kit (Thermo Fisher Scientific, 23225). Protein samples (50–100 μg) were resolved by SDS/PAGE and transferred onto PVDF membranes (Millipore) for following probing with antibodies. The immune-reactive proteins were visualized by enhanced chemiluminescence detection system (Millipore).

### Immunoprecipitation

For Complex I and II analysis, 10 cm dish cultured cells were harvested and ruptured in ice-cold IP lysis buffer (20 mM HEPES (pH 7.4), 0.5% Triton X-100, 150 mM NaCl, 12.5 mM β-glycerophosphate, 1.5 mM MgCl_2_, 2 mM EGTA, NaF 10 mM, Na_3_VO_4_ 1 mM, containing protease and phosphatase inhibitors) for 30 min. Gentle vortex was needed during incubation before clarified by centrifugation at 13,000 *g* for 15 min. An aliquot (10%) of the supernatant was retained as input, and the remaining was performed for immunoprecipitation. The cell lysates were added with the anti-Flag-beads for Complex I or primary antibody and 50 μl protein A-Sepharose beads (GE) for Complex II to incubate with rocking overnight at 4 °C. The immunocomplexes were harvested next day and washed three times with IP lysis buffer. Proteins combined with the beads were eluted with SDS/PAGE sample buffer and subsequently boiled for western blot analysis. To immunoprecipitate RIP1, cells were lysed with buffer (Tris-HCl 20 mM (pH 7.5), NaCl 150 mM, EDTA 1 mM, EGTA 1 mM, Triton X-100 1%, Glycerol 10%, Protease Inhibitor cocktail, Sodium pyrophosphate 2.5 mM, β-Glycerrophosphate 1 mM, NaVO4 1 mM, Leupeptin 1 μg/ml).

### Statistical analysis

All experiments were conducted using 3–15 mice or repeated three independent times with cells. Two-tailed Student’s *t* test or analysis of variance (ANOVA) was used for assessment of all experiments, unless stated otherwise. Data are presented as the mean ± SEM. *P* value < 0.05 was considered statistically significant.

## Results

### Bcl-3 is upregulated by TNF in the liver

**Fig. 1:**
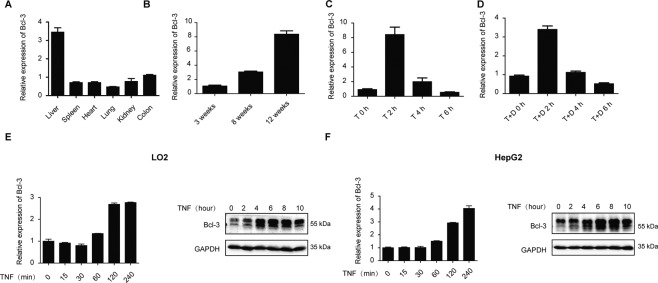
TNF-induced upregulation of Bcl-3 in the liver. **A** qPCR analysis of *bcl-3* in different organs of wild-type mice. **B** qPCR analysis of *bcl-3* in livers from wild-type mice at different ages. **C** qPCR analysis of *bcl-3* expression in the livers following treatment with TNF. **D** qPCR analysis of *bcl-3* expression in the liver following treatment with TNF and D-GalN. **E** Bcl-3 expression was analyzed by qPCR and western blotting in LO2 cells. **F** Bcl-3 expression was analyzed by qPCR and western blotting in HepG2 cells. Abbreviations are as follows: T, TNF (20 mg/kg); T + D, TNF (10 mg/kg) +D-GalN (700 mg/kg).

To assess the role of Bcl-3 in the liver, we first performed a tissue survey detecting the expression of Bcl-3 in various wild-type mouse tissues by RT-qPCR. We observed extraordinarily higher expression of Bcl-3 in the liver than in other organs (Fig. 1A). Moreover, we found that the expression of Bcl-3 was age-dependent and reached a peak at the age of 12 weeks (Fig. 1B). In vivo stimulation with TNF significantly upregulated the transcript level of Bcl-3 in the liver at 2 h (Fig. 1C). Likewise, co-stimulation with TNF and D-GalN also revealed that Bcl-3 was markedly induced within 2 h (Fig. 1D). Moreover, in vitro stimulation of the hepatic cell lines LO2 and HepG2 with TNF also showed upregulated mRNA and protein levels of Bcl-3 (Fig. 1E, F). Thus, we propose that Bcl-3 plays a role in the liver in response to TNF stimulation.

### Bcl-3 deficiency protects mice from TNF-induced liver injury

To further investigate the role of Bcl-3, we generated Bcl-3-deficient mice using the CRISPR/Cas9 system (Fig. S1A, B). We found that Bcl-3 expression in the liver, spleen and colon was completely inhibited in Bcl-3 KO mice (Fig. S1C). Furthermore, we also detected increases in spleen size and percentages of the marginal zone of B cells in Bcl-3 KO mice, as previously reported [30] (Fig. S1D, E).

**Fig. 2:**
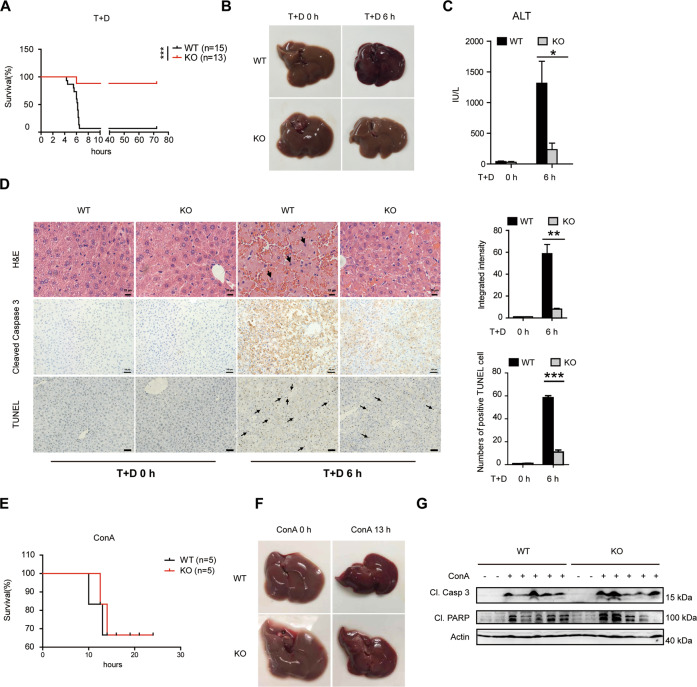
Bcl-3 deficiency leads to decreased hepatoxicity and lethality in response to TNF in vivo. Ten- to 12-week-old male WT and Bcl-3 KO mice were injected intraperitoneally with T + D. **A** Survival was monitored every 2 h for 3 days. **B** Liver tissues were collected and photographed at 6 h after injection. **C** Levels of serum ALT (IU/L) in WT and KO mice treated with PBS or T + D. **D** Hepatic tissues were collected after injection and subjected to histological analysis using H&E staining (scale bar = 20 μm) and immunohistochemistry with anti-cleaved Caspase 3 antibody and TUNEL staining (scale bar = 100 μm). The black arrows denote hepatocytes with fragmented nuclei. The frequency of the integrated intensity of cleaved Caspase 3^+^ or TUNEL^+^ hepatocytes is shown in the graphs on the right. **E** Survival curve of WT and KO mice injected intravenously with ConA. **F** Liver tissues were collected and photographed at 13 h after injection. **G** Western blot analysis of cleaved Caspase 3 and PARP in liver tissues after ConA treatment for 13 h. The results are shown as the mean ± SEM. *p < 0.05, **p < 0.01, ***p < 0.001.

Next, TNF/D-GalN was employed to examine the function of Bcl-3 during liver injury. Bcl-3 KO mice and WT controls were intraperitoneally injected with D-GalN and TNF, and the survival rate was subsequently monitored for three days. We found that Bcl-3 KO mice showed dramatically reduced lethality, while the majority of WT mice died four to 6 h after the injection (Fig. 2A). Because hepatotoxicity serves as the pivotal contributor to lethality in this model, we also observed severe haemorrhage in WT mice administrated TNF/D-GalN but not in KO mice (Fig. 2B). Consistent with this observation, serum alanine transaminase (ALT) activity, which is a hepatotoxic marker, was significantly lower in the KO group after TNF/D-GalN treatment (Fig. 2C). Moreover, histopathological examination with haematoxylin and eosin (H&E) staining showed that intensive liver damage, including extensive swelling of hepatocytes and intralobular haemorrhage, appeared in WT liver tissues 6 h after TNF/D-GalN stimulation, while these effects were mild in KO mice (Fig. 2D). Cleaved Caspase 3 staining and the TUNEL assay (Fig. 2D) also provided strong evidence that less liver injury and fewer apoptotic hepatocytes occurred in Bcl-3-deficient livers due to the hepatotoxic effect of TNF.

As plant lectin concanavalin A (ConA) is another widely used mouse model for introducing immune-mediated acute hepatitis by inducing TNFα, IL-6 and IFNγ [31], we further detected the role of Bcl-3 in ConA-induced liver injury. After intravenous injection of ConA, both WT and Bcl-3 KO mice died from ten to twelve hours and there was no statistic difference in survival between these two groups (Fig. 2E). Meanwhile, we observed severe liver haemorrhage (Fig. 2F) in both groups with ConA treatment and detected significant activation of caspase cascade (Fig. 2G) by immunoblotting. Collectively, these results suggest that Bcl-3 plays a unique role in TNF-induced liver injury and Bcl-3 deficiency significantly protects mice against TNF/D-GalN-induced hepatotoxicity and lethality.

### Bcl-3 regulates the hepatic lethal effect of TNF in a caspase-dependent manner

**Fig. 3:**
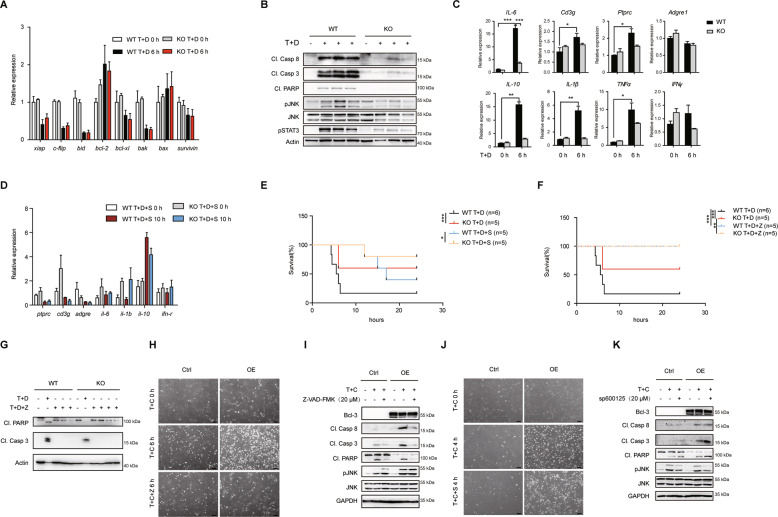
The attenuated sensitivity to TNF-induced cell death in Bcl-3-deficient mice relies on caspase activity. **A** qPCR analysis of NF-κB target genes associated with survival and apoptosis. **B** Cell lysates were prepared from liver tissues 6 h post injection and probed by immunoblotting. **C** qPCR analysis of inflammation markers and immune cell infiltration markers in either WT or KO liver tissues with T + D injection. **D** qPCR analysis of inflammation markers and immune cell infiltration markers in liver tissues from mice with 20 mg/kg sp600125 pretreatment followed by T + D injection. **E** Survival rates of WT and KO mice with T + D or T + D + S treatment. **F** Survival rates of WT and KO mice with or without 10 mg/kg Z-VAD-FMK pretreatment followed by T + D injection. **G** Western blot analysis of cleaved Caspase 3 and PARP in liver tissues from mice with indicated treatment at 10 h. **H** Bcl-3 overexpressing and control MEFs were photographed by phase contrast microscopy after pretreatment with or without 20 μM Z-VAD-FMK as indicated (scale bar = 50 μm). **I** Western blot analysis of caspase and JNK activity as in (**H**). **J** Bcl-3 overexpressing and control MEFs were photographed after pretreatment with or without 20 μM sp600125 followed by T + C treatment as indicated (scale bar = 50 μm). **K** Western blot analysis of caspase and JNK activity as indicated. The results are shown as the mean ± SEM. *p < 0.05, **p < 0.01, ***p < 0.001. Abbreviations are as follows: T + D + S, TNF + D-GalN+sp600125; T + D + Z, TNF + D-GalN+Z-VAD-FMK; T + C, TNF (10 ng/ml) +CHX (10 μg/ml); T + C + Z, TNF + CHX + Z-VAD-FMK; T + C + S, TNF + CHX + sp600125.

It is well known that TNF activates the NF-κB, JNK or caspase cascade to induce either hepatocyte survival or apoptosis; thus, we next characterized the individual roles of these pathways. First, we examined the livers of the indicated groups for the expression of NF-κB-targeted genes that correlate with survival, including *xiap*, *c-flip*, *bid*, *bcl-2*, *bcl-xl*, *bak*, *bax* and *survivin*. The results showed that the expression of these genes was suppressed by D-GalN, a transcriptional inhibitor, but the difference between WT and KO mice was not statistically significant regardless of the administration strategy (Fig. 3A). However, immunoblotting analysis demonstrated exceptionally higher expression levels of cleaved Caspase 8, Caspase 3 and PARP in WT livers than in KO livers after stimulation (Fig. 3B), indicating excessive activation of the caspase cascade. Moreover, we detected enhanced JNK activation in WT livers (Fig. 3B), which further validated the previous report that JNK activation is associated with liver injury [13]. Thus, it is necessary to further assess the impact of caspase and JNK activation on Bcl-3-regulated TNF-induced apoptosis. Given that inflammation arises simultaneously with acute liver injury, Bcl-3 KO livers treated with TNF/D-GalN also showed milder inflammation than WT controls as determined by the expression of NF-κB or JNK targeted inflammation genes such as *IL-6*, *Cd3g*, *Ptprc*, *Adgre 1*, *IL-10*, *IL-1β*, *TNFα* and *IFNγ* (Fig. 3C). Since D-GalN specifically inhibits the activation of NF-κB, we speculate that JNK-induced inflammation may play a role in TNF/D-GalN-induced liver injury. Although pretreatment with JNK inhibitor sp600125 suppressed the expression of TNF/D-GalN-induced inflammation genes (Fig. 3D), it cannot eliminate the difference in death between WT and KO mice after TNF/D-GalN injection (Fig. 3E). However, pretreatment with the caspase inhibitor Z-VAD-FMK in vivo entirely inhibited TNF-induced liver injury in Bcl-3 KO and WT mice (Fig. 3F) by suppressing caspase activation (Fig. 3G). Moreover, in Bcl-3-overexpressing MEFs, we observed more apparent cell death than controls after treatment with TNF and the translation inhibitor cycloheximide (CHX) (Fig. 3H, J). Pretreatment with Z-VAD-FMK almost completely inhibited TNF/CHX-induced apoptosis and eliminated differences in apoptosis between Bcl-3 OE MEFs and controls (Fig. 3H). Western blotting also showed that the activation of caspase cascade had no visible differences between these two groups treated with Z-VAD (Fig. 3I). However, pretreatment with the sp600125 had no effect on the suppression of TNF/CHX-induced apoptosis in Bcl-3 OE MEFs (Fig. 3J). Immunoblotting analysis showed increased levels of cleaved Caspase 8, Caspase 3 and PARP in Bcl-3 OE group treated with sp600125 (Fig. 3K).

Collectively, these findings indicate that Bcl-3 regulates TNF-induced hepatic apoptosis through the caspase cascade rather than NF-κB or JNK signalling.

### Decreased sensitivity to TNF-induced liver injury in Bcl-3-deficient mice is hepatocyte-intrinsic

**Fig. 4:**
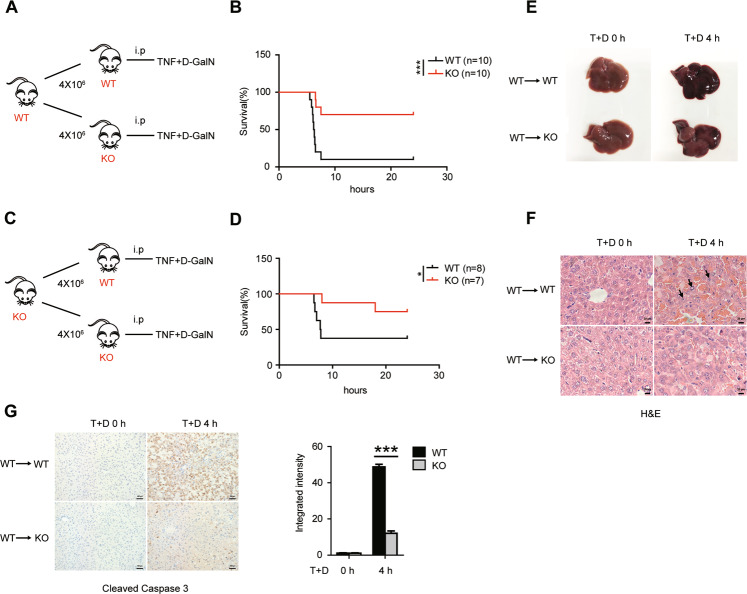
The reduced sensitivity to TNF in Bcl-3-deficient mice is independent of lymphocytes. **A** Schematic showing that lethally irradiated WT and KO mice transplanted with wild-type bone marrow cells were injected intraperitoneally with T + D. **B** Survival of WT and KO recipients treated with T + D was monitored every 2 h for 2 days. **C** Schematic showing that lethally irradiated WT and KO mice transplanted with Bcl-3-deficient bone marrow were injected with T + D. **D** Survival of WT and KO recipients was monitored. **E** Liver tissues were collected and photographed at 4 h after injection. **F** H&E staining of livers of the recipients 4 h post injection (scale bar = 20 μm). **G** Immunohistochemical analysis of livers of the recipients were stained for cleaved Caspase 3 at 4 h after injection (scale bar = 100 μm). The integrated intensity is shown on the right. The results are shown as the mean ± SEM. *p < 0.05, **p < 0.01, ***p < 0.001.

As described above, decreased liver injury occurred concomitantly with reduced inflammation and lymphocyte infiltration in Bcl-3 KO mice. We next characterized the contribution of hematopoietic cells in this model. To this end, we examined the survival rate of TNF/D-GalN-treated recipient WT and Bcl-3 KO mice, which were lethally irradiated and pre-transferred with bone marrow cells from WT donors (Fig. 4A). The results showed an increased survival rate in recipient Bcl-3 KO mice (Fig. 4B). Using a similar approach, we also observed a similar result when Bcl-3 KO mice were used as donors (Fig. 4C, D). Moreover, the pathological morphology exhibited more severe haemorrhage in the WT recipients after 4 h of treatment with TNF/D-GalN (Fig. 4E). In addition, H&E staining revealed enhanced swelling and rupture of hepatocytes in WT recipients (Fig. 4F). Immunochemical analysis of cleaved Caspase 3 in different liver tissues also showed significantly increased apoptotic hepatocytes in WT recipients (Fig. 4G). These data suggest that the distinguishable sensitivity to TNF-induced liver injury between WT and Bcl-3 KO mice is hepatocyte-intrinsic rather than hematopoietic cell-dependent.

### Decreased apoptosis induced by TNF in Bcl-3-deficient hepatic cells

**Fig. 5:**
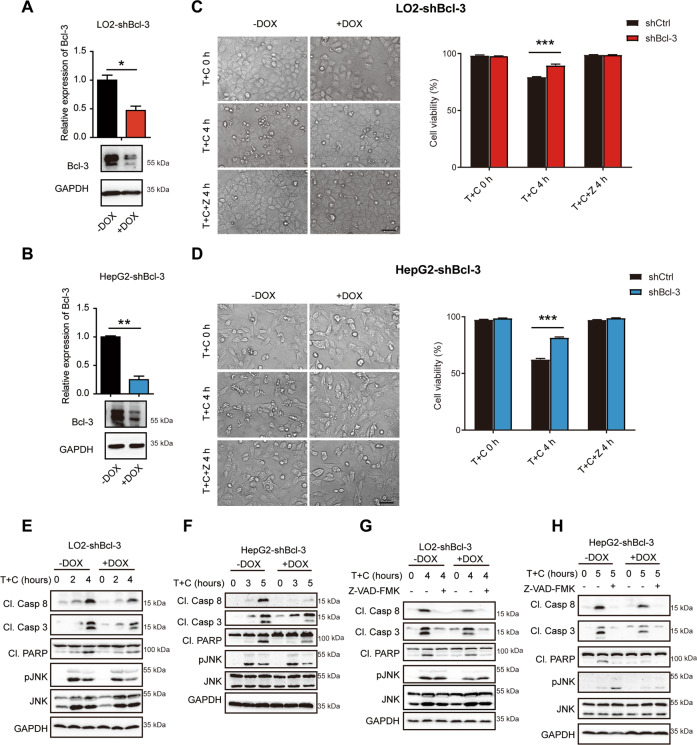
Bcl-3 knockdown desensitizes LO2 and HepG2 cells to TNF/CHX-induced apoptosis. **A** Bcl-3-knockdown efficiency in tet-on stable LO2 cells upon DOX treatment was quantified by qPCR and western blotting. **B** Bcl-3 knockdown efficiency was analyzed in tet-on stable HepG2 cells upon DOX treatment. **C** LO2-shBcl-3 cells were treated with the indicated stimulations and then photographed (scale bar = 50 μm) and analyzed by flow cytometry for Annexin V/7-AAD staining. **D** HepG2-shBcl-3 cells were treated with the indicated stimulations and then photographed (scale bar = 50 μm) and analyzed by flow cytometry for Annexin V/7-AAD staining. **E** Western blot analysis of caspase and JNK activation in Bcl-3 knockdown LO2 cells with T + C treatment. **F** Western blot analysis of caspase and JNK activation in Bcl-3 knockdown HepG2 cells with T + C treatment. **G** LO2-shBcl-3 cells were treated with or without Z-VAD-FMK and analyzed by western blot. **H** HepG2-shBcl-3 cells were treated with or without Z-VAD-FMK and analyzed by western blot. The results are shown as the mean ± SEM. *p < 0.05, **p < 0.01, ***p < 0.001.

To further validate the in vivo experiments, we characterized the role of Bcl-3 in TNF-induced cell apoptosis in vitro. Two hepatic cell lines, HepG2 and LO2, were used for further investigation of the underlying mechanism. Given the high expression level of Bcl-3 in hepatic cells, we established Bcl-3 knockdown cell lines using a doxycycline (DOX)-inducible tet-on system or Bcl-3 KO cells by CRISPR/Cas9 system. Tet-on LO2 and HepG2 cells were treated with 1 μg/ml DOX for more than three days to downregulate Bcl-3 at the mRNA and protein levels (Fig. 5A, B). We next assessed the sensitivity of Bcl-3 knockdown or KO cells and the controls in response to TNF/CHX-induced apoptosis. Cellular morphology and flow cytometric analysis of apoptotic cells showed a protective role of Bcl-3 deletion from TNF-induced apoptosis (Fig. 5C, D, S5). Immunoblotting analysis also showed obviously decreased expression of cleaved Caspase 8, Caspase 3 and PARP and slightly decreased JNK activation in Bcl-3 knockdown or KO cells (Fig. 5E, F, S5). Moreover, pretreatment with Z-VAD-FMK successfully inhibited TNF/CHX-induced apoptosis and eliminated intergroup differences in both cell lines (Fig. 5C, D). Western blotting analysis also confirmed this result (Fig. 5G, H). Moreover, knockdown of Bcl-3 with another shRNA sequence in these two cell lines exhibited the same results (Fig. S2). Apart from Bcl-3 knockdown cells, Bcl-3 overexpressing NIH3T3 cells also exhibited the similar results (Fig. S3).

The in vitro data strongly support that Bcl-3 deficiency plays a pivotal role in protecting against TNF-induced apoptosis.

### Bcl-3 promotes complex II formation by deubiquitinating RIP1

Collectively, the in vivo and in vitro data unveiled a novel role of Bcl-3 in regulating TNF-induced apoptosis and liver injury. To further investigate the underlying mechanism, we first detected the influence of Bcl-3 on TNFR1 expression, which is the initial molecule in TNF signalling. We found that Bcl-3 deficiency had no effect on TNFR1 expression in vivo after TNF/D-GalN treatment (Fig. S4A) or in tet-on HepG2 and LO2 cells stimulated with TNF/CHX (Fig. S4B, C). Similarly, the expression levels of TNFR1 in Bcl-3 OE NIH3T3 cells and controls with indicated treatment were not significantly different (Fig. S4D). Herein, we confirmed that TNFR1 expression was completely unaffected by Bcl-3.

**Fig. 6:**
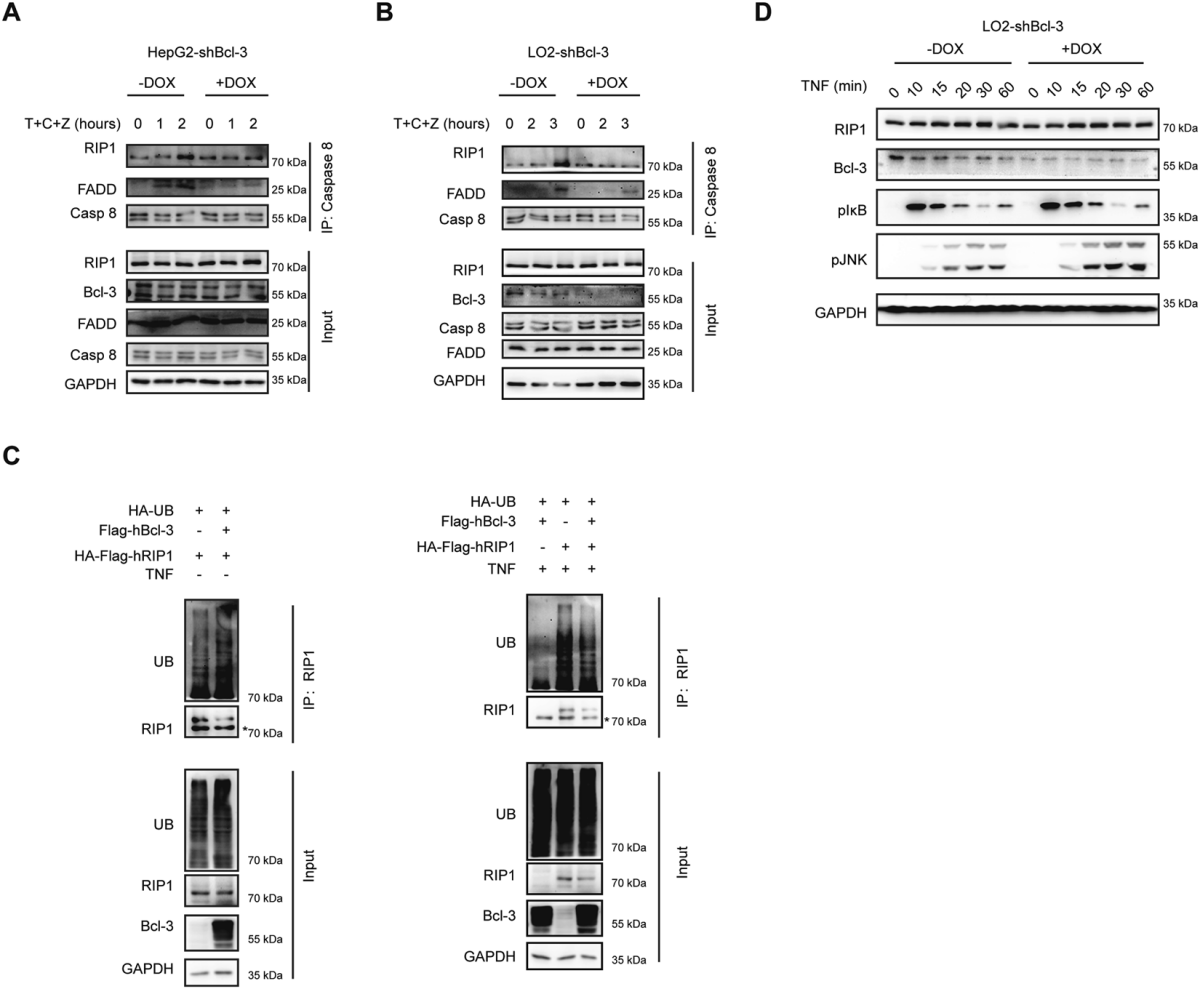
Bcl-3 regulates RIP1 ubiquitination to form Complex II. **A** Lysates of HepG2-shBcl-3 cells treated with T + C + Z at the indicated times were immunoprecipitated with anti-Caspase 8 antibody. The immunoprecipitated proteins were probed with the indicated antibodies. **B** Lysates of LO2-shBcl-3 cells treated with T + C + Z were immunoprecipitated with anti-Caspase 8 antibody. **C** 293 T cells were transfected with HA-Flag-RIP1 and HA-UB, together with the empty control vector or Flag-Bcl-3 expression vector. Cell lysates were immunoprecipitated with anti-RIP1 antibody after treatment with or without TNF for 20 min. **D** LO2-shBcl-3 cells were stimulated with TNF for the indicated times, followed by western blot analysis of pIκB and p-JNK.

It is well known that Caspase 8, the central cell death regulator, is directly activated by Complex II; thus, we hypothesized that Bcl-3 plays a role in regulating the formation of Complex II in response to TNF and D-GalN or CHX. To assess this hypothesis, we conducted coimmunoprecipitation experiments. In tet-on HepG2 cells, cell lysates were treated with the indicated stimulations and immunoprecipitated with Caspase 8, and disrupted time-dependent interactions with FADD and RIP1 to form Complex II were detected in Bcl-3 knockdown cells (Fig. 6A). Moreover, a similar result was observed in tet-on LO2 cells (Fig. 6B). Increasing evidence suggests that RIP1 is predominantly deubiquitinated in Caspase 8-associated Complex II. Thus, we hypothesized that Bcl-3 promotes the deubiquitination of RIP1. In support of this hypothesis, we revealed that the TNF-induced ubiquitination of RIP1 in 293 T cells overexpressing both Bcl-3 and RIP1 was obviously lower than that in cells overexpressing only RIP1 (Fig. 6C), indicating that Bcl-3 promotes the deubiquitination of RIP1. It is known that TNF-induced ubiquitination of RIP1 is essential for the activation of JNK signalling. Herein, we also found that Bcl-3 deficiency greatly activated the JNK cascade, as increased expression level of p-JNK was observed in Bcl-3-knockdown LO2 cells, which further confirmed the role of Bcl-3 in regulating the deubiquitination of RIP1 (Fig. 6D).

### Bcl-3 regulates RIP1 deubiquitination mainly in a CYLD-dependent manner

**Fig. 7:**
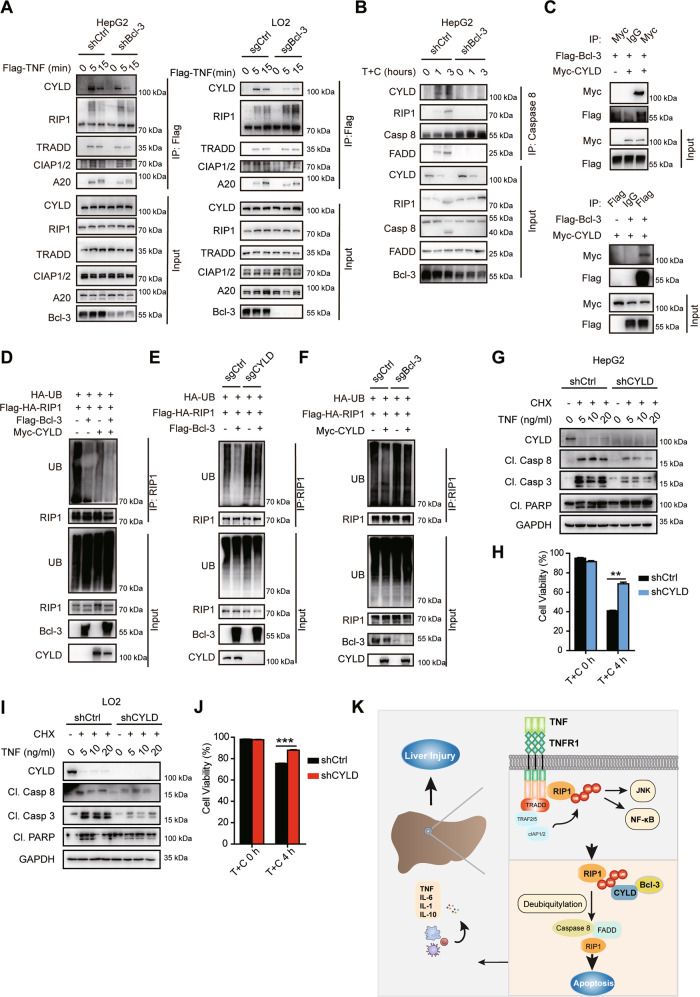
RIP1 deubiquitination regulated by Bcl-3 is CYLD-dependent. **A** Bcl-3-deficient and control cells were stimulated with 500 ng/ml Flag-TNF for indicated times to induce Complex I formation. The immunocomplex were analyzed by western blotting. **B** Bcl-3-deficient and control cells stimulated with T + C stimuli as indicated to promote Complex II formation. The immunocomplex were analyzed by western blotting. **C** 293 T cells were transfected with Bcl-3 and CYLD, and cell lysates were immunoprecipitated with anti-Myc or anti-Flag antibody together with control IgG antibody after 48 h. The immunocomplex was analyzed by immunoblotting. **D** 293 T cells transfected with UB, RIP1 together with CYLD, Bcl-3 or empty control vector were immunoprecipitated with anti-RIP1 antibody after TNF treatment for 20 min. **E** CYLD knockout and control 293 T cells transfected with UB、RIP1 with or without Bcl-3 were immunoprecipitated with anti-RIP1 antibody after 20 ng/ml TNF stimulation. **F** Bcl-3 knockout and control 293 T cells transfected with UB、RIP1 with or without CYLD were immunoprecipitated with anti-RIP1 antibody after 20 ng/ml TNF stimulation. **G** Western blot analysis of caspase activation in CYLD-knockdown and control HepG2 cells with the indicated treatments. **H** Cell viability of CYLD-knockdown and control HepG2 cells stimulated with T + C was analyzed by Annexin V/7-AAD staining. **I** Western blot analysis of caspase activation in CYLD-knockdown and control LO2 cells with the indicated treatments. **J** Cell viability of CYLD-knockdown and control LO2 cells stimulated with T + C was analyzed by Annexin V/7-AAD staining. **K** Schematic model for the role of Bcl-3 in TNF-induced hepatic apoptosis. The results are shown as the mean ± SEM. *p < 0.05, **p < 0.01, ***p < 0.001.

Ubiquitination of RIP1 has been reported to be regulated by cIAPs, deubiquitinase CYLD or A20 in Complex I or II [19–21]. In this study, we did not observe the direct interaction of Bcl-3 with RIP1 (Fig. S6). As there is no predicted enzyme domain in Bcl-3, we speculated that Bcl-3 might exert its function by association with such components. Then we checked the effects of Bcl-3 on key deubiquitinases in Complex I and II upon TNF stimulation. Ablation of Bcl-3 significantly reduced the amount of CYLD rather than A20 or cIAPs in TNF-induced Complex I (Fig. 7A) and II (Fig. 7B) formation, consequentially resulting in decreased binding of RIP1 and FADD in Complex II (Fig. 7B). As Bcl-3 has been reported to bind to the USP domain harboring the DUB activity of CYLD [32–34], we hypothesize that Bcl-3 may regulate RIP1 deubiquitination in a CYLD-dependent manner. We examined the interaction between Bcl-3 and CYLD as reported in 293 T cells by co-IP assay (Fig. 7C). We further found that transfection of RIP1 with either Bcl-3 or CYLD evidently promoted RIP1 deubiquitination (Fig. 7D). Moreover, Bcl-3-induced RIP1 deubiquitination can be largely rescued by KO of CYLD (Fig. 7E) and the loss of Bcl-3 similarly suppressed the deubiquitination activity of CYLD (Fig. 7F). In conclusion, these results indicated that Bcl-3 could regulate the binding of CYLD into the RIP1 containing Complex I and II and thus facilitate RIP1 deubiquitination in the context of TNF. In addition to CYLD, the results also suggest that there are unrevealed mechanisms by which Bcl-3 regulates RIP1 deubiquitination.

Moreover, we showed that downregulation of CYLD in either HepG2 or LO2 cells resulted in reduced levels of Caspase activity (Fig. 7G, I). Annexin V/7-AAD staining also showed that knockdown of CYLD set cells resistant to TNF/CHX-induced cell apoptosis (Fig. 7H, J). Taken together, our results indicated that Bcl-3 functioned in upregulating TNF-induced hepatic apoptosis by facilitating the removal of poly-ub from RIP1 in a CYLD-dependent way, thus promoting the formation of death-inducing complex II (Fig. 7K).

## Discussion

TNF-induced cytotoxicity has been implicated in various liver diseases, including alcoholic liver disease and chronic viral hepatitis, as well as acute liver injury [35]. Increasing evidence suggests that TNF-triggered pathways leading to hepatocyte death serve as attractive targets for therapeutic intervention in liver diseases. Therefore, we attempted to find new targets for liver diseases caused by TNF-induced hepatotoxicity. In this study we found that Bcl-3 promotes TNF-induced hepatocyte apoptosis by enhanced RIP1 deubiquitination, providing a novel Bcl-3-targeted strategy for various liver diseases.

As described previously, we and others found that Bcl-3 is highly expressed in mouse liver tissues (Fig. 1A) and human livers (BioGPS database), but whether and how Bcl-3 plays a role in liver injury is still unknown. Here, by taking advantage of Bcl-3 KO mice, we assessed the role of Bcl-3 in liver injury. Surprisingly, coadministration of TNF and the hepatocyte-specific transcriptional inhibitor D-GalN resulted in greater reduced lethality in Bcl-3 KO mice, indicating a novel role of Bcl-3 in TNF-induced liver injury. Previous reports indicated that TNF-induced liver injury is caused by hepatocyte apoptosis and inflammation-induced cell necrosis with rapid recruitment of neutrophils [35, 36]. Here, we also detected reduced neutrophil infiltration and inflammation in Bcl-3 KO mice with TNF/D-GalN treatment. Subsequently, we assessed the role of Bcl-3 in inflammation-induced liver injury after TNF stimulation by using bone marrow transfer assays. When Bcl-3 component bone marrow cells were transferred from wild-type mice, the recipient Bcl-3 KO mice still showed less lethality, indicating the decreased sensitivity to TNF-induced hepatotoxicity after Bcl-3 depletion is hepatocyte-intrinsic rather than lymphocyte-dependent. Consistent with previous findings, we suggest that hepatocytes are primarily sensitive to the lethal effect of TNF in the presence of transcription or translation inhibitors, including D-GalN and CHX. The in vitro data also provided evidence that Bcl-3 deficiency protects cells from TNF-induced apoptosis, which strongly supports the in vivo conclusion.

In addition, we detected a lower level of IL-6 expression and STAT3 activation in Bcl-3 KO mice (Fig. 3B, C) and knockdown cells (Fig. S2G, H) in response to TNF-induced cytotoxicity. Previous reports indicated that IL-6/STAT3 activation plays a crucial role in the repair of defective hepatocytes and liver regeneration [37, 38] by upregulating the expression of a series of target cytokines and growth factors. Accordingly, the decreased IL-6/STAT3 activation in Bcl-3-deficient mice is consistent with reduced liver injury, suggesting Bcl-3 might regulates both the liver injury and regeneration.

TNF is well known to cause cell death as well as survival by NF-κB activation, which is essential for the expression of hepatoprotective genes. In this study, NF-κB activation was almost completely inhibited by D-GalN or CHX, and TNF mainly acted as a death activator. Thus, we reasoned that the insusceptibility to the lethal effect of TNF after Bcl-3 deficiency is NF-κB-independent. Moreover, multiple pathways activated by TNF have been shown to induce cell death in the liver, especially the caspase cascade and JNK signalling. To assess the specific roles of Bcl-3 in these individual pathways, JNK and caspase inhibitors were used for further investigation, and the results showed that decreased sensitivity to TNF-induced cytotoxicity in the absence of Bcl-3 relies on the caspase cascade rather than JNK signalling. Furthermore, due to the extremely low expression level of RIP3 in hepatocytes [39], we suggest that no obvious necroptosis induced by TNF occurred in this work.

TNF-induced caspase activity is directly regulated by the formation of Complex II, in which the ubiquitination status of RIP1 plays as the crucial step. In this study, we demonstrated that Bcl-3 promotes RIP1 deubiquitination in a CYLD-dependent manner (Fig. 7K). As *Miyazaki and Korneluk* [40, 41] reported that CYLD plays an indispensable role in promoting TNF-induced Complex II formation in the presence of smac-mematics, we also detected the role of Bcl-3 in regulating TNF and smac-mematics LCL161-induced hepatic cell death. We found that knockdown of Bcl-3 dramatically inhibited caspase activity (Fig. S7). These data revealed that Bcl-3 might promote the formation of both Complex I and Complex II through regulating CYLD participation in complex formation.

In this study, we showed an NF-κB-independent role of Bcl-3 in the liver, and our work suggests deletion of Bcl-3 greatly ameliorates TNF-induced liver injury, which provides a potential therapeutic target for various liver diseases. Moreover, we showed that Bcl-3 exhibits no effect on FasL or TraiL-induced apoptosis in either HepG2 or LO2 cells (Fig. S8). We also investigated the role of Bcl-3 in the response to TNF stimulation in other cell types, including colon cancer cells and breast cancer cells. Conversely, Bcl-3 exerted a protective effect against TNF/CHX-induced apoptosis in these cells (data not shown). Thus, our data indicate that Bcl-3 plays an unique role in the hepatocytes apoptosis and liver injury induced by TNF.

Bcl-3 can be induced by various liver injury risk factors, such as LPS [42], HBV [43], alcohol [44] and high fat diet [45]. Increased Bcl-3 might promote TNF-induced apoptosis in hepatocytes after challenge with the liver injury risk factors by facilitating the deubiquitination of RIP1 mediated by CYLD. Therefore, our work revealed that Bcl-3 could serve as a potential therapeutic target for treating abnormal hepatocyte apoptosis and liver injury.

## Supplementary information

Supplementary Figure and Table legends

supplementary Figure 1

supplementary Figure 2

supplementary Figure 3

supplementary Figure 4

supplementary Figure 5

supplementary Figure 6

supplementary Figure 7

supplementary Figure 8

Supplementary Table

Author contribution

Reproducibility Checklist

## Data Availability

All the data used in the current study are available from the corresponding author on reasonable request.
