# Supplementary material for: Bcl-3 promotes TNF-induced hepatocyte apoptosis by regulating the deubiquitination of RIP1
Source: Cell Death Differ. 2021 Dec 1;29(6):1176–86. doi: 10.1038/s41418-021-00908-7 (PMC9177694; doi:10.1038/s41418-021-00908-7)
Supplement: Supplementary file 1 — Supplementary Figure and Table legends [file 41418_2021_908_MOESM1_ESM.docx]

**Supplementary information**

**Supplementary Figure Legends**

**Supplementary 1. Construction of Bcl-3-deficient mice by using CRISPR/Cas9.**

(A) Generation of mice with targeted disruption of exon 2 of the *bcl-3* gene locus. The magnified views illustrate the binding sites of the sgRNAs. F and R represent the forward and reverse primers used in genotyping, respectively. (B) Genotyping of Bcl-3-deficient and WT mice by PCR analysis of genomic DNA. (C) qPCR analysis of *bcl-3* expression in the liver, spleen and colon of Bcl-3-deficient and wild-type control mice. (D) Spleens of Bcl-3-deficient and WT mice were collected and photographed. (E) Marginal zone B cells were collected from the spleens of Bcl-3-deficient and WT mice and analyzed by flow cytometry. The results are shown as the mean ± SEM. *p < 0.05, ** p < 0.01, *** p < 0.001.

**Supplementary 2. Bcl-3 knockdown desensitizes LO2 and HepG2 cells to TNF/CHX-induced apoptosis.**

The second inducible shBcl-3 sequence was used to deplete Bcl-3 in LO2 and HepG2 cells. (A) Bcl-3 knockdown efficiency was examined in LO2 cells by qPCR and western blotting. (B) Caspase and JNK activation in LO2-shBcl-3 and control cells treated with T+C was detected by immunoblotting. (C) Caspase and JNK activation in LO2-shBcl-3 and control cells pretreated with or without Z-VAD-FMK was detected by immunoblotting. (D) Bcl-3 knockdown efficiency was examined in HepG2 cells. (E) Caspase and JNK activation in HepG2-shBcl-3 cells treated with T+C was detected by immunoblotting. (F) Caspase and JNK activation in HepG2-shBcl-3 and control cells pretreated with or without Z-VAD-FMK was detected by immunoblotting. (G) qPCR analysis of *IL-6* in LO2-shBcl-3 cells treated with T+C at the indicated times. (H) qPCR analysis of *IL-6* in HepG2-shBcl-3 and control cells treated with T+C at the indicated times. The results are shown as the mean ± SEM. *p < 0.05, ** p < 0.01, *** p < 0.001.

**Supplementary 3. Bcl-3 overexpressing NIH3T3 cells show increased apoptosis in response to TNF/CHX co-stimulation.**

NIH3T3 cells were infected with lentivirus to construct stable Bcl-3-overexpressing cell lines. (A) Bcl-3 overexpressing and control NIH3T3 cells were photographed (scale bar=50 μm) after treatment with T+C or T+C+Z. (B) Western blot analysis of caspase and JNK activation after T+C treatment in NIH3T3 cells. (C) Western blot analysis of caspase and JNK activity in NIH3T3 cells after T+C+Z treatment. (D) Flow cytometry analysis with Annexin V/7-AAD staining of NIH3T3 cells after T+C or T+C+Z treatment. (E) The percentages of Annexin V^+^ and 7-AAD^+^ cells are shown. The results are shown as the mean ± SEM. *p < 0.05, ** p < 0.01, *** p < 0.001.

**Supplementary 4. Bcl-3 has no effect on the expression of *TNFR1.***

(A) qPCR analysis of *TNFR1* in the liver tissues of WT and Bcl-3-deficient mice treated with T+D at the indicated times. (B) qPCR analysis of *TNFR1* in Bcl-3 knockdown and control HepG2 cells treated with T+C at the indicated times. (C) qPCR analysis of *TNFR1* in Bcl-3 knockdown and control LO2 cells treated with T+C at the indicated times. (D) qPCR analysis of *TNFR1* in Bcl-3 overexpressing and control NIH3T3 cells treated with T+C at indicated times.

**Supplementary 5. Bcl-3 ablation desensitizes LO2 cells to TNF/CHX-induced apoptosis.**

(A) Western blot analysis of caspase activation in Bcl-3 knockout LO2 cells with 20 ng/ml TNF and 10 μg/ml CHX treatment. (B) Annexin V/7-AAD staining for LO2 cells treated with TNF/CHX as indicated.

**Supplementary 6. Bcl-3 has no interaction with RIP1.**

293T cells were transfected with RIP1 or Bcl-3 or both as indicated, and after 20 ng/ml TNF stimulation, cell lysates were immunoprecipitated with anti-HA antibody. The immunocomplex was analyzed by immunoblotting.

**Supplementary 7. Bcl-3 decreased TNF/LCL161-induced hepatic cell death.**

(A) LO2 and HepG2 were stimulated with 20 ng/ml TNF and 10 μM LCL161 for 12 hours or 24 hours. cIAP1/2 and caspase activity were analyzed by western blot. (B) Western blot analysis of caspase activity in LO2-shBcl-3 and control cells stimulated with 20 ng/ml TNF and 5 μM or 10 μM LCL161 for 24 hours.

**Supplementary 8. Bcl-3 has no effect on FasL or TraiL-induced hepatic cell death.**

(A) HepG2-shBcl-3 and control cells were treated with the indicated stimulations and analyzed by flow cytometry for cleaved Caspase 3 staining. (B) The percentages of cleaved Caspase 3^+^ cells are shown in HepG2 cells treated with FasL or TraiL and CHX. (C) Caspase activation in HepG2 cells treated with the indicated stimulations was detected by immunoblotting. (D) The percentages of cleaved Caspase 3^+^ cells analyzed by flow cytometry are shown in LO2 cells treated with FasL or TraiL and CHX. (E) Caspase activation in LO2 cells treated with the indicated stimulations was detected by immunoblotting.

**Supplementary Table Legend**

**Supplementary table 1. Real-time PCR Primers.**
