# Supplementary figures and images for: Bcl-3 promotes TNF-induced hepatocyte apoptosis by regulating the deubiquitination of RIP1
Source: Cell Death Differ. 2021 Dec 1;29(6):1176–86. doi: 10.1038/s41418-021-00908-7 (PMC9177694; doi:10.1038/s41418-021-00908-7)

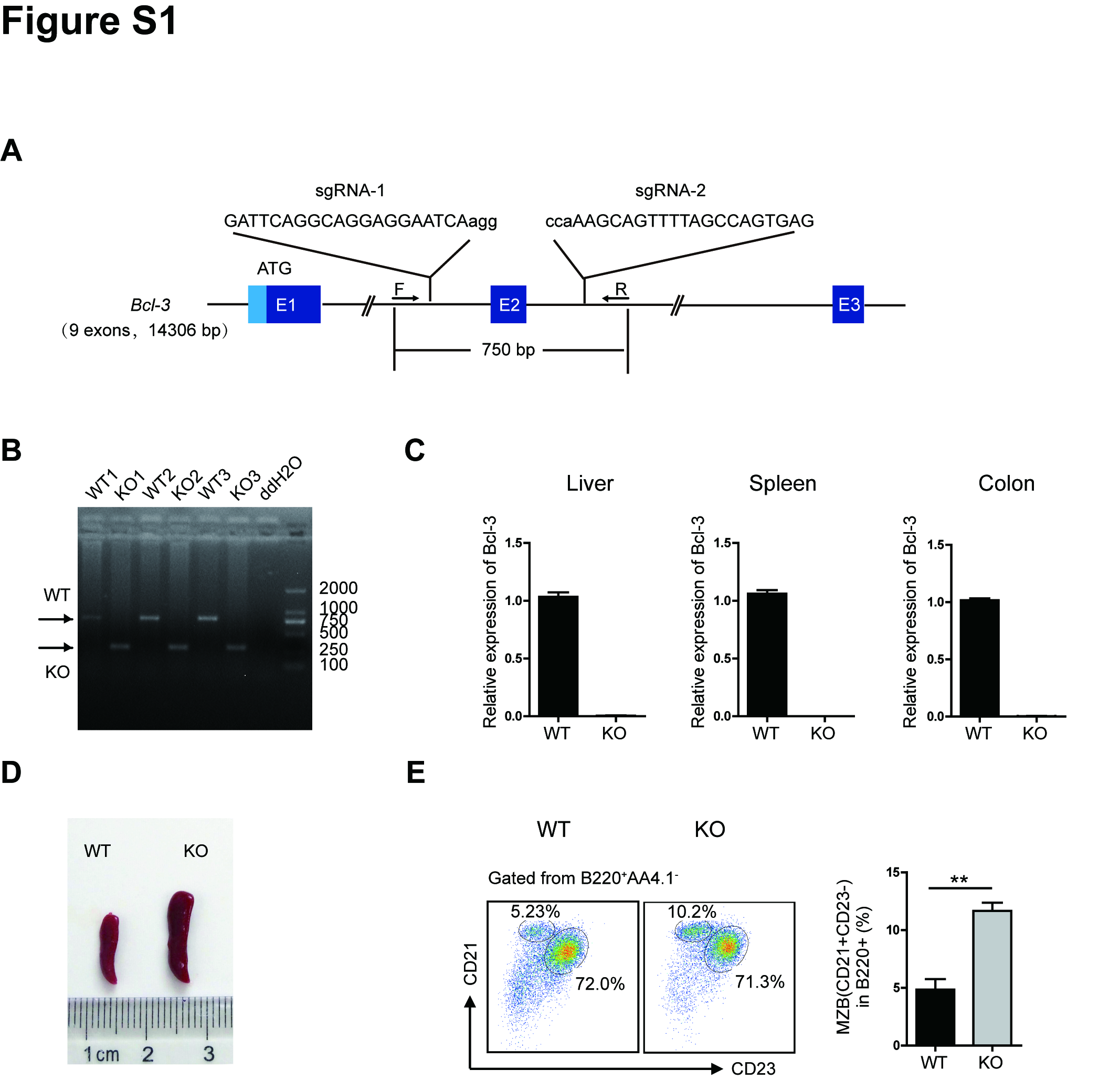

Supplement: Supplementary file 2 — supplementary Figure 1 [file 41418_2021_908_MOESM2_ESM.tif]

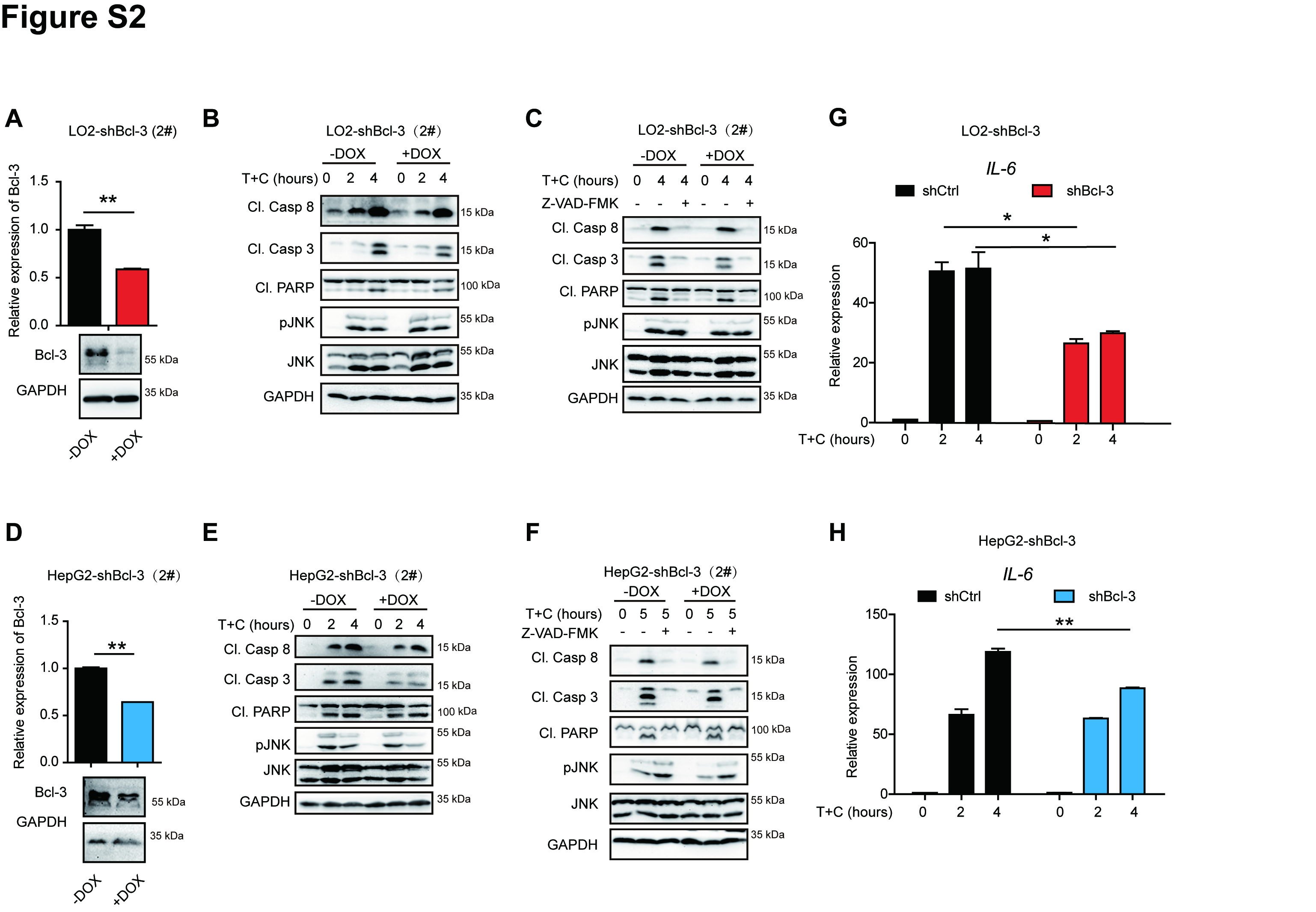

Supplement: Supplementary file 3 — supplementary Figure 2 [file 41418_2021_908_MOESM3_ESM.tif]

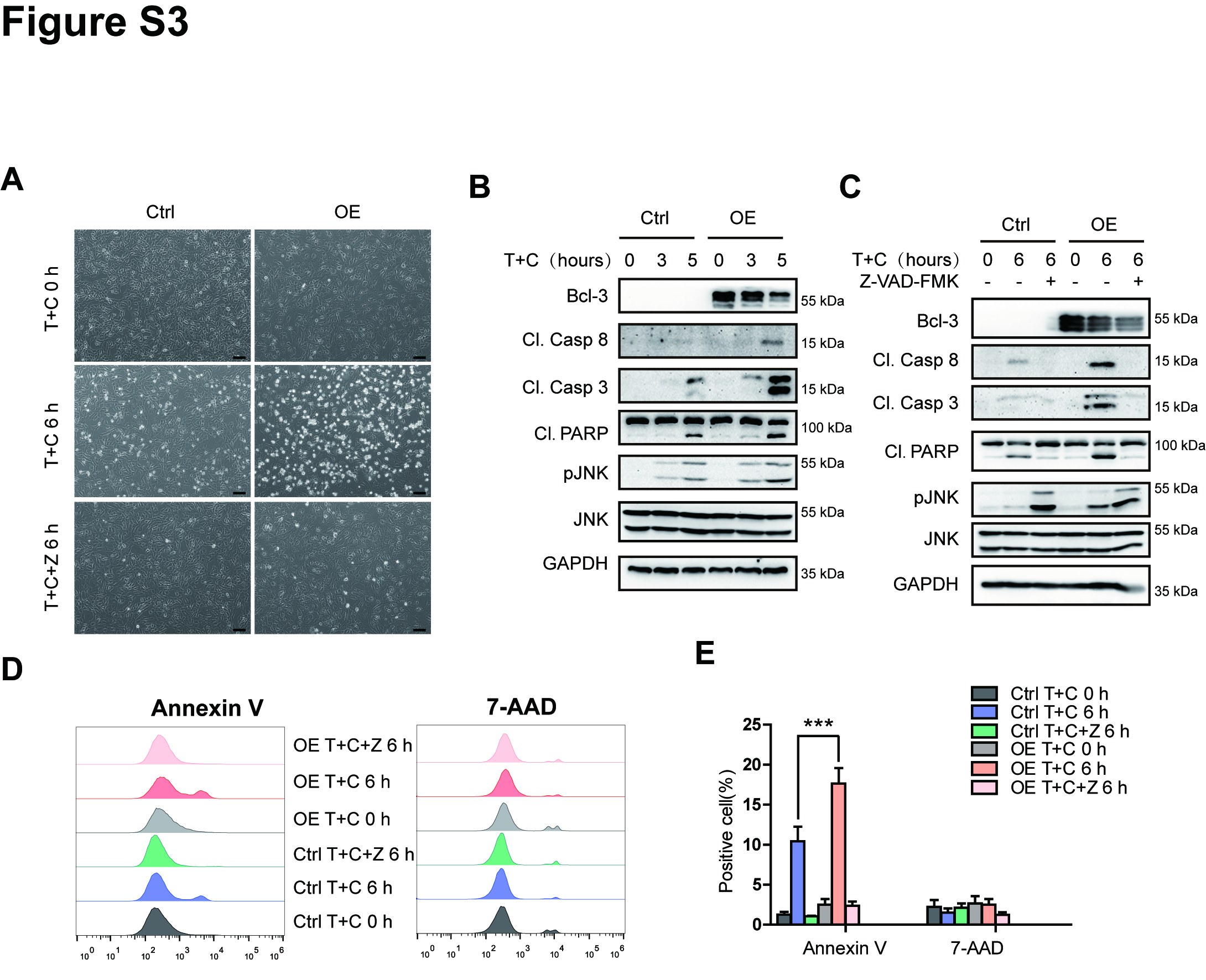

Supplement: Supplementary file 4 — supplementary Figure 3 [file 41418_2021_908_MOESM4_ESM.tif]

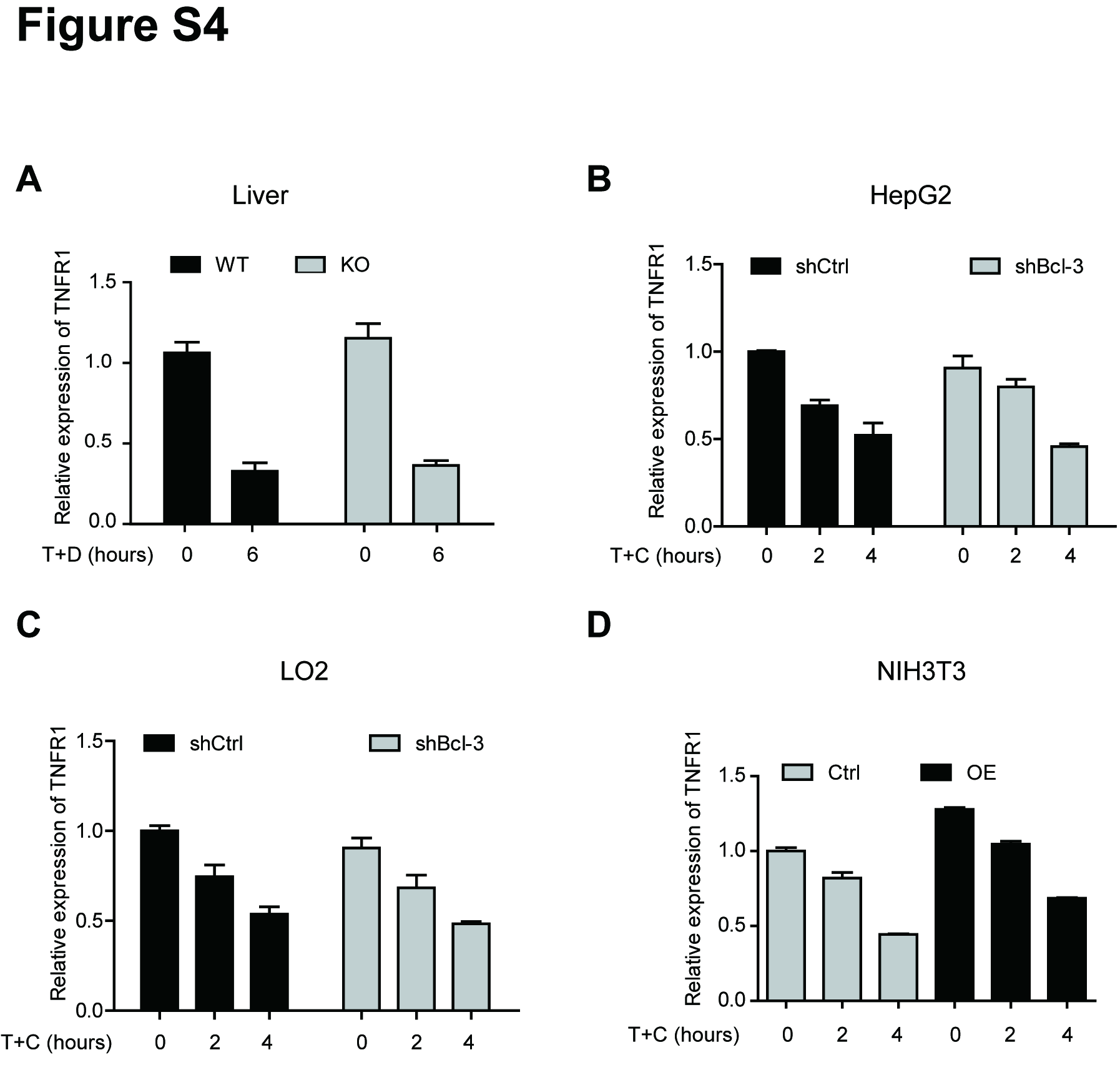

Supplement: Supplementary file 5 — supplementary Figure 4 [file 41418_2021_908_MOESM5_ESM.tif]

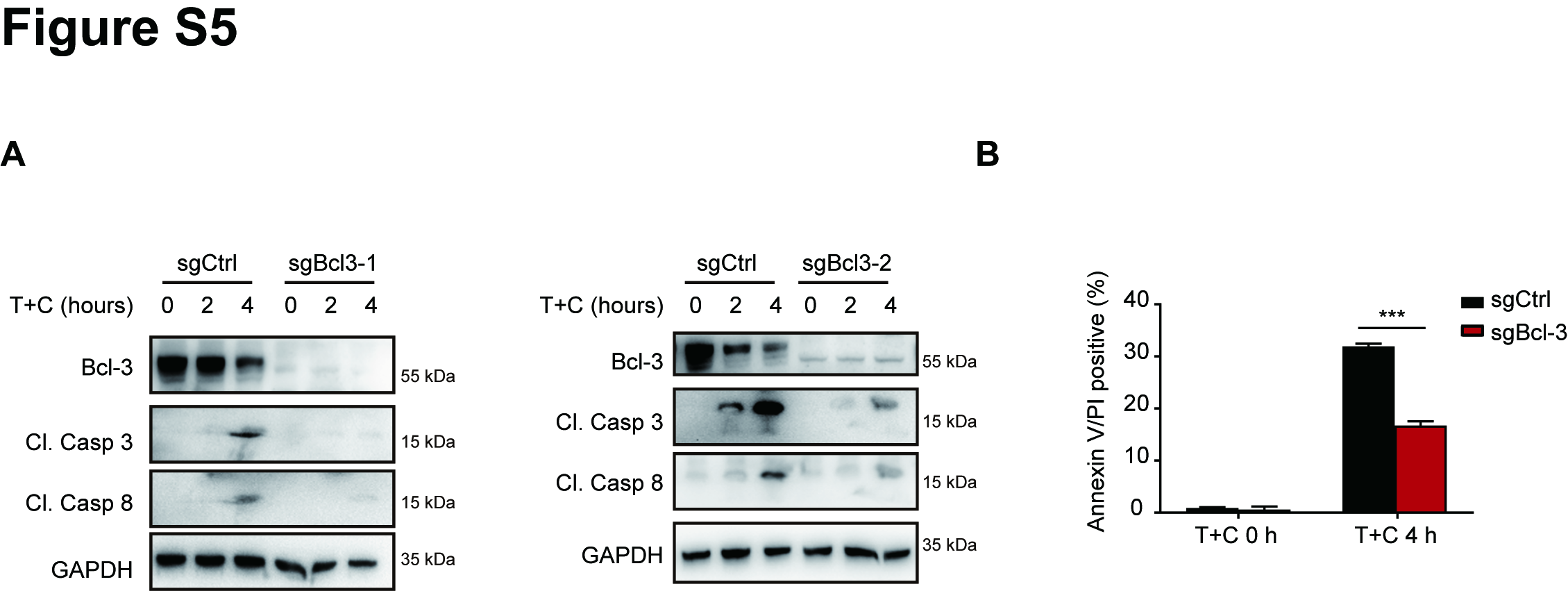

Supplement: Supplementary file 6 — supplementary Figure 5 [file 41418_2021_908_MOESM6_ESM.tif]

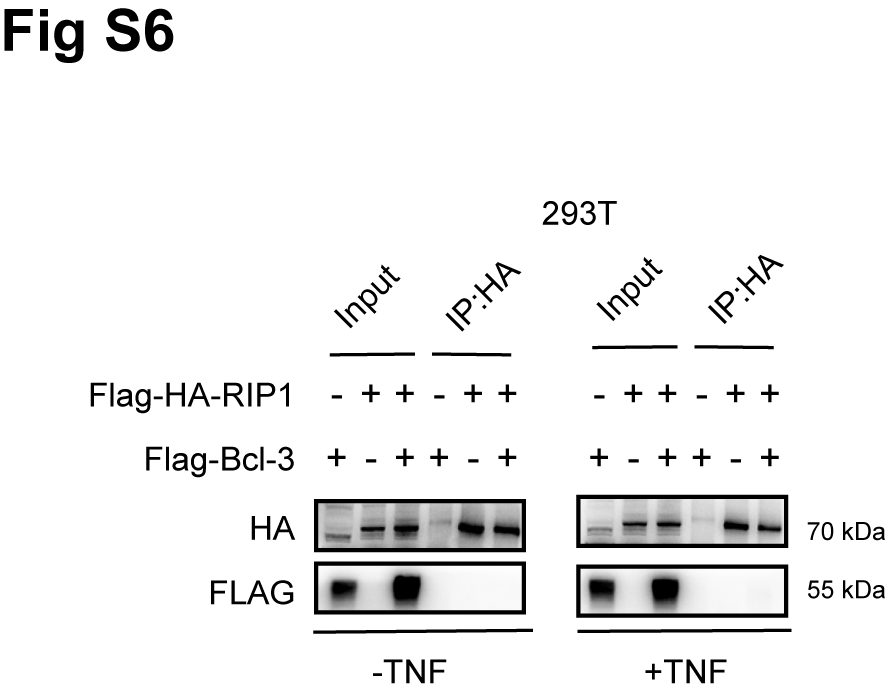

Supplement: Supplementary file 7 — supplementary Figure 6 [file 41418_2021_908_MOESM7_ESM.tif]

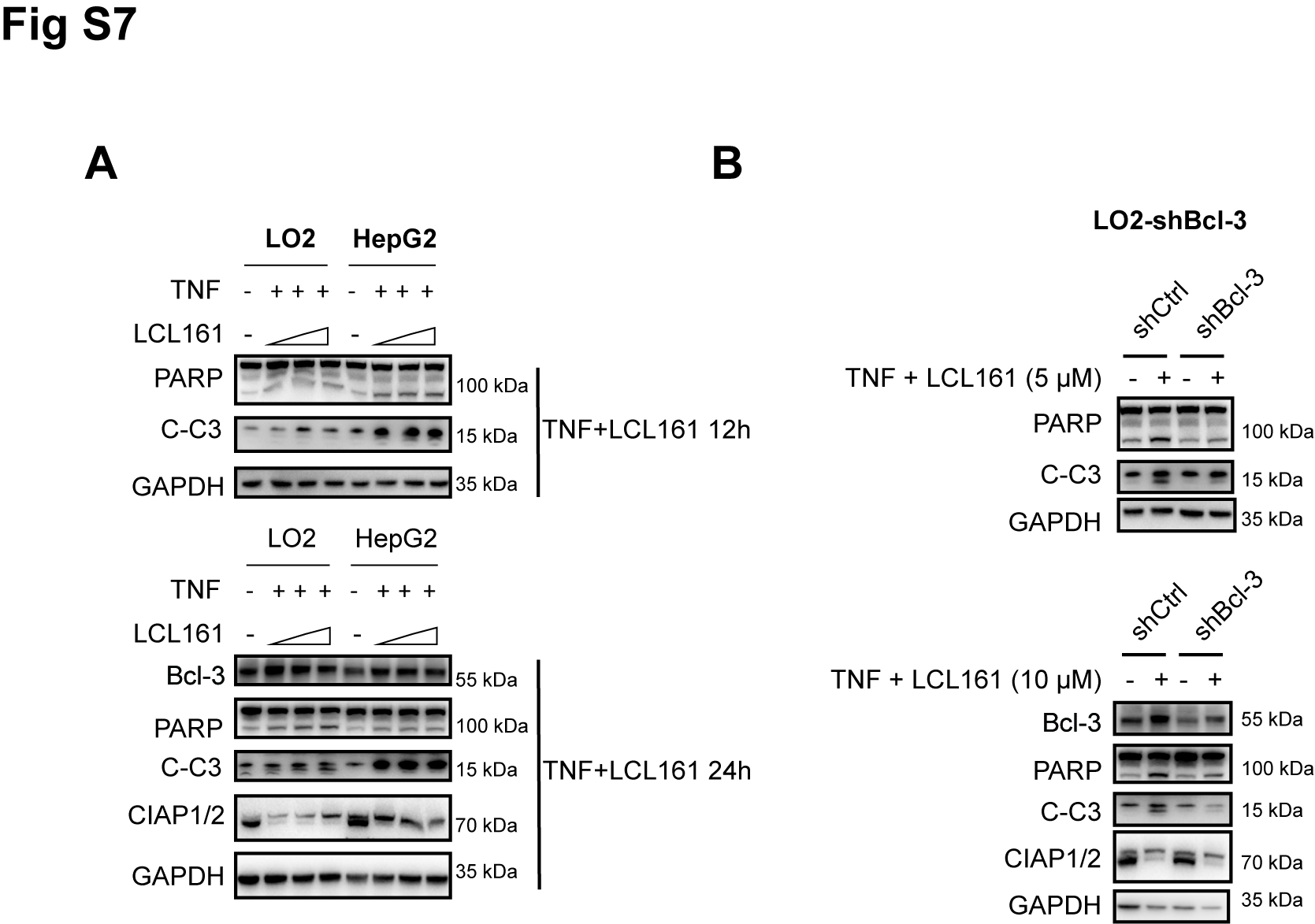

Supplement: Supplementary file 8 — supplementary Figure 7 [file 41418_2021_908_MOESM8_ESM.tif]

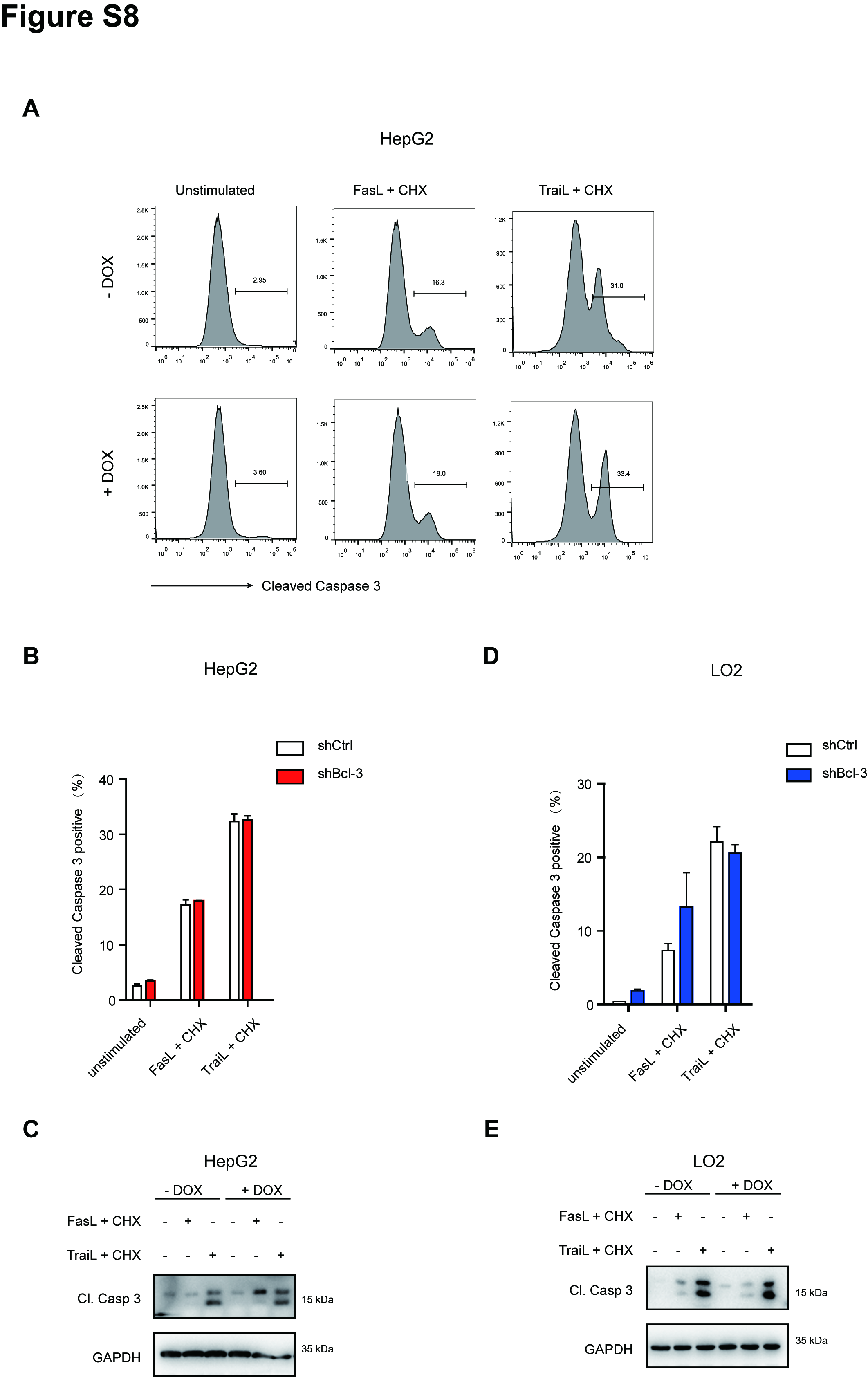

Supplement: Supplementary file 9 — supplementary Figure 8 [file 41418_2021_908_MOESM9_ESM.tif]
