# Supplementary material for: Bcl-3 promotes TNF-induced hepatocyte apoptosis by regulating the deubiquitination of RIP1
Source: Cell Death Differ. 2021 Dec 1;29(6):1176–86. doi: 10.1038/s41418-021-00908-7 (PMC9177694; doi:10.1038/s41418-021-00908-7)
Supplement: Supplementary file 11 — Author contribution [file 41418_2021_908_MOESM11_ESM.pdf]

DECLARATION OF CONTRIBUTIONS TO ARTICLE

ADMC

Manuscript Number: CDD-20-1155R

Journal Name: *Cell Death & Differentiation* (the 'Journal')

Proposed Title of the Contribution: Author contributions (the 'Contribution)

Author(s): Yiming Hu, Haohao Zhang, Ningxia Xie, Dandan Liu, Yuhang Jiang, Zhi Liu, Deji Ye, Sanhong Liu, Xi Chen, Cuifeng Li, Qi Wang, Xingxu Huang, Yongzhong Liu, Yufang Shi, Xiaoren Zhang (the 'Authors')

For all *CDD* articles, each person named as an author in the published version must be able to show he or she has contributed substantially to the article.

Authorship credit should be based on 1) substantial contributions to conception and design, acquisition of data, or analysis and interpretation of data; 2) drafting the article or revising it critically for important intellectual content; and 3) final approval of the version to be published. Authors should meet conditions 1, 2 and 3.

Any person who cannot be shown to have made a substantial contribution to the article cannot be listed as an author in the final version. The name of any person who is deemed to have made a minor contribution can, however, appear in the Acknowledgments section of the article.

Please complete the table below to indicate the contributions of all named authors to the manuscript.

Author Full Name:	Specification of Contribution to the Manuscript:
Yiming Hu	Conception and design; Acquisition of data; Analysis and interpretation of data; Drafting the article
Haohao Zhang	Acquisition of data; Analysis of data; Revising the article
Ningxia Xie	Conception and design; Revising the article
Dandan Liu	Acquisition of data
Yuhang Jiang	Acquisition of data
Zhi Liu	Acquisition of data
Deji Ye	Acquisition of data
Sanhong Liu	Acquisition of data
Xi Chen	Acquisition of data
Cuifeng Li,Qi Wang	Acquisition of data
Xingxu Huang, Yongzhong Liu	Revising the article
Yufang Shi	Conception and design; Revising the article critically for important intellectual content
Xiaoren Zhang	Conception and design; Analysis of data; Revising the article; Final approval of the version to be published

Please complete the table below to indicate the contributions of all named authors to the figures.

Figure 1:

Y.H. and H.Z. collected the liver tissues and conducted qPCR analysis. H.Z. and D.L. stimulated cells and conducted western blotting analysis. Y.H. assembled the figure.

Figure 2:

Y.H. and H.Z. conducted TNF+D-GalN related experiments. Y.J. performed immunohistochemical analysis. Z.L. was responsible for detecting ALT. D.Y. conducted ConA related experiments. Y.H. assembled the figure.

Figure 3:

Y.H. and H.Z. conducted all the qPCR experiments and in vivo experiments. Y.H. performed western blotting analysis. S.L. photographed cells with indicated stimulation. Y.H. assembled the figure.

Figure 4:

Y.H. and H.Z. conducted the BMT experiments and collected liver tissues for HE and immunohistochemical detection. X.C. was responsible for statistics analysis. Y.H. assembled the figure.

Figure 5:

Y.H. and H.Z. conducted all the western blot analysis. C.L. performed flow cytometry analysis. Q.W. was responsible for statistics analysis and photographed the cells with stimulation. Y.H. assembled the figure.

Figure 6:

Y.H. conducted western blotting for Fig. A-C. H.Z. performed analysis for Fig. D. Y.H. assembled the figure.

Signed for and on behalf of the Author(s):	Print Name:	Date:
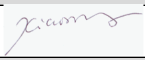	Xiaoren Zhang	2021.2.26
